# *Ceratophyllum demersum* the submerged macrophyte from the mining subsidence reservoir Nadrybie Poland as a source of anticancer agents

**DOI:** 10.1038/s41598-024-57375-6

**Published:** 2024-03-20

**Authors:** Maciej Masłyk, Tomasz Lenard, Marta Olech, Aleksandra Martyna, Małgorzata Poniewozik, Anna Boguszewska-Czubara, Elżbieta Kochanowicz, Paweł Czubak, Konrad Kubiński

**Affiliations:** 1 Department of Molecular Biology, The John Paul II Catholic University of Lublin, Ul. Konstantynów 1I, 20-708 Lublin, Poland; 2 Department of Animal Physiology and Toxicology, The John Paul II Catholic University of Lublin, Ul. Konstantynów 1I, 20-708 Lublin, Poland; 3 Department of Pharmaceutical Botany, Medical University of Lublin, Ul. Chodźki 1, 20-093 Lublin, Poland; 4 Department of Plant Physiology and Biotechnology, The John Paul II Catholic University of Lublin, Ul. Konstantynów 1I, 20-708 Lublin, Poland; 5 Chair and Department of Medical Chemistry, Medical University of Lublin, Ul. Chodźki 4a, 20-093 Lublin, Poland

**Keywords:** Cancer, Drug discovery, Ecology, Plant sciences

## Abstract

Aquatic plants are a rich source of health-beneficial substances. One of such organisms is the submerged macrophyte *Ceratophyllum demersum*, which has not been sufficiently studied in this aspect so far. In this work, we have studied environmental conditions prevailing in a subsidence mining reservoir in Eastern Poland and shown that *C. demersum* can be harvested for further analysis even from artificial anthropogenic reservoirs. The phytochemical analysis of *C. demersum* ethanolic extract using LC–MS revealed high content of phenolic compounds (18.50 mg/g) (mainly flavonoids, 16.09 mg/g), including those that have not yet been identified in this plant, namely isorhamnetin, sakuranetin, taxifolin, and eriodictyol. Such rich flavonoid content is most likely responsible for the anticancer activity of the *C. demersum* extract, which was targeted especially at neoplastic cells of gastrointestinal tract origin. The flow cytometry analysis of treated cells showed an increased percentage of late apoptotic and necrotic cells. The fish embryo toxicity (FET) test showed safety of the extract towards *Danio rerio* fish up to the concentration of 225 µg/ml. This study has shown that the submerged macrophyte *Ceratophyllum demersum* can be taken into consideration as a rich source of a set of anticancer agents with chemopreventive potential.

## Introduction

Plants produce many biologically active substances, i.e., amino acids, lipids, carbohydrates, alkaloids, glycosides, flavonoids, polyphenols, polysaccharides, and many others^1,2^. All bioactive compounds are generally divided into two groups: primary and secondary metabolites. While primary metabolites are used for growth and development, secondary metabolites allow plants to interact with the surrounding environment. This gives them an advantage over other species occupying the same ecological niche^3^. These substances can act on any component of the natural environment, e.g. bacteria, fungi, plants, and animals, including humans^1,2^. The effect of plant extracts on living organisms and the application of the extracts in medical therapies are being widely investigated^2^. Although the majority of such research is focused on terrestrial plants, aquatic vegetation seems to have potential in such studies as well.

Plant active substances have been considered to have medicinal value for thousands of years. Their use as therapeutic agents is known throughout the world, starting with ancient Indian and Chinese herbalism^2,4,5^. This type of research conducted with advanced modern techniques has been developing nowadays^3,6–8^. The vast majority, however, is focused on terrestrial higher plants. In contrast, still little is known about secondary metabolites of submerged macrophytes from aquatic environments or the impact of these metabolites on human health and their potential application in medicinal therapies.

Aquatic ecosystems demonstrate a high degree of variability in their both abiotic and biotic environmental characteristics. Among others, lakes are expected to be an interesting object of study since they can present two main alternative stable states^9^. As shown by many authors, the state of turbid water dominated by phytoplankton or clear water predominated by aquatic vegetation have a well-established background in research and literature^10–13^. Nevertheless, in the face of the intensifying climate change, the boundaries between these two states have been blurred^14,15^. Aquatic plants are more susceptible to damage by environmental changes than terrestrial plants. However, hornwort (*Ceratophyllum demersum L*.) is an example of a submerged macrophyte that can adapt well to a rapid shift from a clear to turbid water state. *C. demersum*, widespread in Eurasia and North America^16^, is a perennial plant which does not form roots but is attached to the bottom sediments by modified leaves. Additionally, hornwort can play an important role in stabilizing and maintaining a clear water state even at high phosphorus concentrations^13^. Moreover, its high shade tolerance and resistance to disturbance in the water environment allow its highly abundant growth in turbid waters^17,18^. Hence, this species can develop widely also in both natural and artificial shallow water bodies. The developmental success of *C. demersum* is a result of its ability to produce and release chemical compounds that have a negative allelopathic effect on other aquatic plants and planktonic autotrophs, including algae, i.e. diatoms and toxin-producing cyanobacteria^11,13,19–24^. However, *C. demersum* is able to stimulate (positive allelopathy) the growth or affect the morphology of some eukaryotic microorganisms like *Chlorella* and *Scenedesmus*, which was confirmed in laboratory experiments^25–27^. Van Donk and van de Bund also confirmed an increase in the biomass of chlorophytes *Chlorella* and *Chlamydomonas* growing within *Ceratophyllum demersum* clumps^28^. Some researchers investigated the promoting or inhibiting effects of aqueous or acetone extracts from *C. demersum* on root elongation, seed germination, or seedling growth in terrestrial plants^29,30^.

Since it has already been documented that *Ceratophyllum demersum* has an impact on water and terrestrial primary producers at different levels of organization, the aim of our study included (i) characterization of the reservoir from which the plant material was collected, (ii) preparation of crude extract using whole plant biomass, (iii) determination of the content of some metabolites, (iv) determination of the anticancer activity of the extract as well as the mechanism of action, and (v) verification of the ecotoxicity and safety using a zebrafish model.

## Material and methods

### Physical and chemical analysis of Nadrybie reservoir water

To describe the environmental conditions in the Nadrybie reservoir, the study was conducted from April to September in 2019 and 2020. The water samples were taken once a month from the middle part of the reservoir from an area devoid of submerged vegetation. Water samples for analyses were always taken with the use of a Ruttner water sampler (2 dm^3^ capacity) from a depth of 0.5 m and 1 m and poured into a bucket. After mixing, one representative sample was taken for further analysis. In the laboratory, the samples were analyzed with spectrophotometric methods to determine the amount of phytoplankton expressed by the concentration of chlorophyll-*a*^31^ and the concentration of biogenic compounds: total phosphorus (TP), total nitrogen (TN), inorganic phosphorus (P-PO_4_), and nitrogen (N–NH_4_; N–NO_3_) according to methods described by Hermanowicz et al.^32^. In addition, water reaction (pH), electrical conductivity (EC), water transparency as Secchi disk visibility (SD), and water temperature were measured in situ. The chemical and biological data were used to evaluate the trophic status of the Nadrybie reservoir using the trophic state indices TSI (CHL), TSI (TP), and TSI (TN), which were calculated according to the equations described by Carlson^33^ and Kratzer and Brezonik^34^.

### Ceratophyllum demersum biomass collection procedure

In the eastern part of the Nadrybie reservoir, we found an aquatic plant community from the class Potametea, order Potametalia, alliance Potamion, and association Ceratophylletum demersi with almost complete domination of hornwort (*Ceratophyllum demersum*) covering a large area of the water body. The plant association was identified according to the nomenclature proposed by Matuszkiewicz^35^. Fresh samples of hornwort were collected during their maximum vegetation (at the turn of July and August) in 2019 and 2020 with the use of a macrophyte anchor from selected places located in the middle part of the Nadrybie reservoir. This helped us to avoid the presence of floating plants e.g. from the class Lemnetea or the influence of emerged plants e.g. from the class Phragmitetea, which may have been found in the shallower littoral zone located nearby the shoreline. Subsequently, the hornwort specimens were pre-cleaned in situ to remove the largest particles of seston from the plant surface and placed in clean plastic containers. Next, the samples were immediately transferred to the laboratory for further cleaning. In the laboratory, in order to remove as much seston as possible, the plants were gently immersed in distilled water for several times and left in a new portion of fresh distilled water for at least one hour. After this time, the plants were rinsed for the last time and placed in a shaded greenhouse for 72-h pre-drying. The next stage of drying took place in a heated room deprived of sunlight at a temperature of 36 °C and lasted 7 days. The plant material prepared in this way was ground and preserved in a zipper-top poly bag for further analyses.

The study on plants was in accordance with relevant institutional, national, and international guidelines and legislation. Collecting small amounts of plants for scientific purposes from water reservoirs owned by the Republic of Poland does not require any permissions.

### Chemicals

Ethanol and sodium carbonate were purchased from Avantor Performance Materials Poland (Gliwice, Poland). Gallic, *m*-coumaric, *o*-coumaric, *p*-coumaric, caffeic, 5-*O*-caffeoylqunic, ferulic, isoferulic, vanillic, salicylic, 3-hydroxybenzoic, 4-hydroxybenzoic, protocatechuic, syringic, rosmarinic acid, quercitrin, apigenin 7-*O*-glucoside, eriocitrin, taxifolin, 3-*O*-methylquercetin, isorhamnetin, isorhamnetin-3-*O*-glucoside, luteolin, kaempferol, rutin, hyperoside, isoquercetin, sakuranetin, aluminum chloride, and LC–MS grade acetonitrile were purchased from Sigma-Aldrich Chemical Co. (St. Louis, Mo, USA). Folin–Ciocalteu reagent was supplied by Chempur (Piekary Śląskie, Poland). Quercetin was purchased from Fluka (Buchs, Switzerland). Catechin, naringenin 7-*O*-glucoside, eriodictyol, luteolin-7-*O*-glucoside, naringenin 7-*O*-glucoside, luteolin 3,7-diglucoside, nicotiflorin, narcissoside, gentisic, sinapic acid, and myricetin were provided by ChromaDex (Irvine, CA, USA). Astragalin, apigenin, naringenin, kaempferol-3-rutinoside, and tiliroside were supplied by Roth (Karlsruhe, Germany). LC–MS grade water was prepared using a Millipore Direct-Q3 purification system (Bedford, MA, USA).

### Extraction

Extracts were obtained using the accelerated solvent extraction technique (ASE 150 extractor; Dionex Corporation; Sunnyvale, CA, USA). Portions (5 g) of dried and pulverized plant material were mixed (1:1, v/v) with diatomaceous earth and loaded into an extraction cell. Three extraction cycles (15 min each) at 80 °C using 80% (v/v) ethanol were performed for each part of the plant material. The eluates were combined, evaporated in a Heidolph Basis Hei-VAP Value evaporator (Schwabach, Germany), lyophilized in the Free Zone 1 apparatus (Labcono, Kansas City, KS, USA), and weighed. The samples were prepared and analyzed in triplicate. The extract yield was calculated and found to be 108.32 ± 2.63 mg/g dry plant material.

### Determination of total phenolic content (TPC)

The total phenolic content was assayed with the method described in detail by Olech et al.^36^ with a slight modification. The sample (20 µL) was mixed with 20 µL of the Folin–Ciocalteu reagent followed by the addition of 160 µL of sodium carbonate (75 g/L). The absorbance was measured at 760 nm after 20 min using an Infinite Pro 200F microplate reader (Tecan Group Ltd., Männedorf, Switzerland). The results were expressed as gallic acid equivalents.

### Determination of total flavonoid content (TFC)

The total flavonoid content was determined with a modified Lamaison and Carnart method as previously described^36^. The results were expressed as quercetin equivalents.

### LC–ESI–MS/MRM analysis

Simultaneous analysis of phenolic acids and flavonoids was performed using liquid chromatography—mass spectrometry (LC–MS/MS). The method was partly based on previous experiments^36,37^. An Agilent 1200 Series LC system (Agilent Technologies, USA) connected to a 3200 QTRAP triple quadrupole mass analyzer (Sciex, Redwood City, CA, USA) equipped with an electrospray ionization (ESI) ion source (AB Sciex, Redwood City, CA, USA) was used. Separations were carried out on an Eclipse XDB-C18 column (4.6 × 150 mm, 5 μm; Agilent Technologies, USA) maintained at 25 °C. The injection volume, mobile phases, and gradient used were the same as those described by Olech et al.^37^. The ESI interface worked in the following conditions: capillary temperature 500 °C, curtain gas at 30 psi, nebulizer gas at 55 psi, negative ionization mode source voltage − 4500 V. The mass analyzer was operated in the multiple reaction monitoring (MRM) scan mode. All the devices were controlled with the Analyst 1.5 software (Sciex, Redwood City, CA, USA, www.sciex.com). Corresponding standard compounds were tested in the same conditions to generate data and parameters needed for qualitative and quantitative purposes. The optimized LC–MS parameters for the qualitative and quantitative analysis of phenolic compounds are given in Supplementary Tables S1 and S2 in the Supplementary Information.

### Cell culture

Six cancer cell lines derived from organs belonging to digestive system, i.e. Detroit-562 (pharyngeal carcinoma), OE21 (esophageal squamous cell carcinoma), AGS (stomach–gastric adenocarcinoma), Hep G2 (hepatocellular carcinoma), SW480 (Dukes' type B, colorectal adenocarcinoma), and HT-29 (colorectal adenocarcinoma), were obtained from ATCC or Sigma-Aldrich. The cells were cultured in appropriate medium (RPMI or DMEM) supplemented with penicillin (100 U/ml), streptomycin (100 U/ml), and 10% heat-inactivated FBS. The cells were maintained in a humidified atmosphere at 37 °C and 5% CO_2_ and passaged twice before performing an experiment.

### Antiproliferative activity assay

For the antiproliferative activity assay, the cells of each cell line were seeded in 96-well microplates at a density of 2.5 × 10^4^ cells/ml in 100 μl of medium supplemented with 10% heat-inactivated FBS. After 24 h of cell attachment, the plates were washed with 100 μl/well with Dulbecco’s phosphate buffered saline (DPBS) and the cells were treated with different concentrations (1–300 μg/ml final concentration) of the tested extract prepared in fresh FBS-free medium for 24, 48, and 72 h. The extract was dissolved in DMSO in order to prepare stock solutions. During the experiments, the stock solutions were diluted in cell culture medium to reach maximum 0.1% w/v DMSO in the final solution. Each concentration was tested in triplicate. 0.1% DMSO was used as a negative control. In the case of the 48- and 72-h incubation, every 24 h the medium was replaced with a fresh one containing the same amount of the compound in order to maintain its concentration at a constant level.

The antiproliferative activity of the compounds was assessed using the MTT (3-[4,5-dimethylthiazole-2-yl]-2,5-diphenyltetrazolium bromide) assay. After each exposure period, the control medium or the test exposure medium were removed, the cells were rinsed with DPBS, and 100 μl of fresh medium (without FBS or antibiotics) containing 0.5 mg/ml of MTT was added to each well. Subsequently, the plates were incubated for 3 h at 37 °C in a 5% CO_2_ humidified incubator. After the incubation period, the medium was discarded, the cells were washed with 100 μl of DPBS, and 100 μl of DMSO was added to each well to extract the dye. The plate was shaken for 10 min and the absorbance was measured at 570 nm (BioTek Synergy H1 microplate reader). Viability was calculated as the ratio of the mean of optical density (OD)] obtained for each condition to the control condition. Statistical analysis, one-way ANOVA, was performed with the use of GraphPad 9 software (version 9.0.0 for Windows, GraphPad Software, Boston, Massachusetts USA, www.graphpad.com).

### Annexin V/propidium Iodide apoptosis study

The SW480 cells were seeded at 1.2 × 10^6^ cells per dish and treated with 10, 20, 40, and 100 µg of extract for 24 h. 0.1% DMSO was used as a control. After the treatment, the cells were trypsinized, centrifuged at 500×*g* for 5 min at room temperature, washed with PBS, and subjected to flow cytometry. Induction of apoptosis was determined by flow cytometry using the FITC Annexin V Apoptosis Detection Kit (BD Pharmingen) following the instructions of the manufacturer. Briefly, the cells were collected and washed twice with cold PBS and then resuspended in 500 µl of 1× Binding Buffer (BD Pharmingen, kit component) at a concentration of 1 × 10^6^ cells/ml. Afterward, 100 µl of the cell suspension was transferred to a 5 ml culture tube and the cells were stained with 5 µl of FITC Annexin V and 5 µl PI. Then, the cells were incubated for 15 min at room temperature in the absence of light and examined by flow cytometry (BD FACS Calibur flow cytometer).

### Zebrafish toxicity studies

To determine the toxicity of the extracts, the fish embryo toxicity (FET) test was performed on zebrafish (*Danio rerio*) according to OECD Test Guideline 236. Briefly, newly fertilized zebrafish eggs were exposed to the tested substances for 96 h at concentrations ranging from 12.5 to 300 μg/ml. The system applied in the experiment was static, as the changes in the solution concentrations did not exceed the range of 20% of nominal concentration values.

The E3 solution (5 mmol/L NaCl, 0.17 mmol/L KCl, 0.33 mmol/L CaCl_2_, and 0.33 mmol/L MgSO_4_, containing no methylene blue, with a pH value of about 7.2) was used as embryo culture medium and to prepare the solutions. The experiment was performed in 24-well plates, with five embryos per well, i.e. ten embryos per group. Each plate was covered and kept in an incubator set at 28 ± 0.5 °C under a light/dark photoperiod of 12/12 h. At the end of the exposure period (96 hpf—hours post fertilization), acute toxicity was determined based on a positive outcome in any of the four visual indicators of lethality, including the coagulation of fertilized eggs, lack of somite formation, lack of detachment of the tailbud from the yolk sac, and lack of heartbeat. The value of LC_50_ was calculated. Additionally, the heartbeat was measured to evaluate the potential cardiotoxicity of the extracts. Statistical analysis, one-way ANOVA, was performed with the use of GraphPad 9 software (version 9.0.0 for Windows, GraphPad Software, Boston, Massachusetts USA, www.graphpad.com).

The experiments with use of *Danio rerio* model was performed on larvae up to 120 h post fertilization, therefore no permission of Ethical Committees was required (acc. to Polish law and EU directives). All experiments with use of in vivo model were performed while respecting the 3R principle and other guidelines (e.g. ARRIVE) for working with animals.

## Results and discussion

### Characteristics of the Nadrybie water reservoir as a source of *Ceratophyllum demersum* biomass

**Figure 1:**
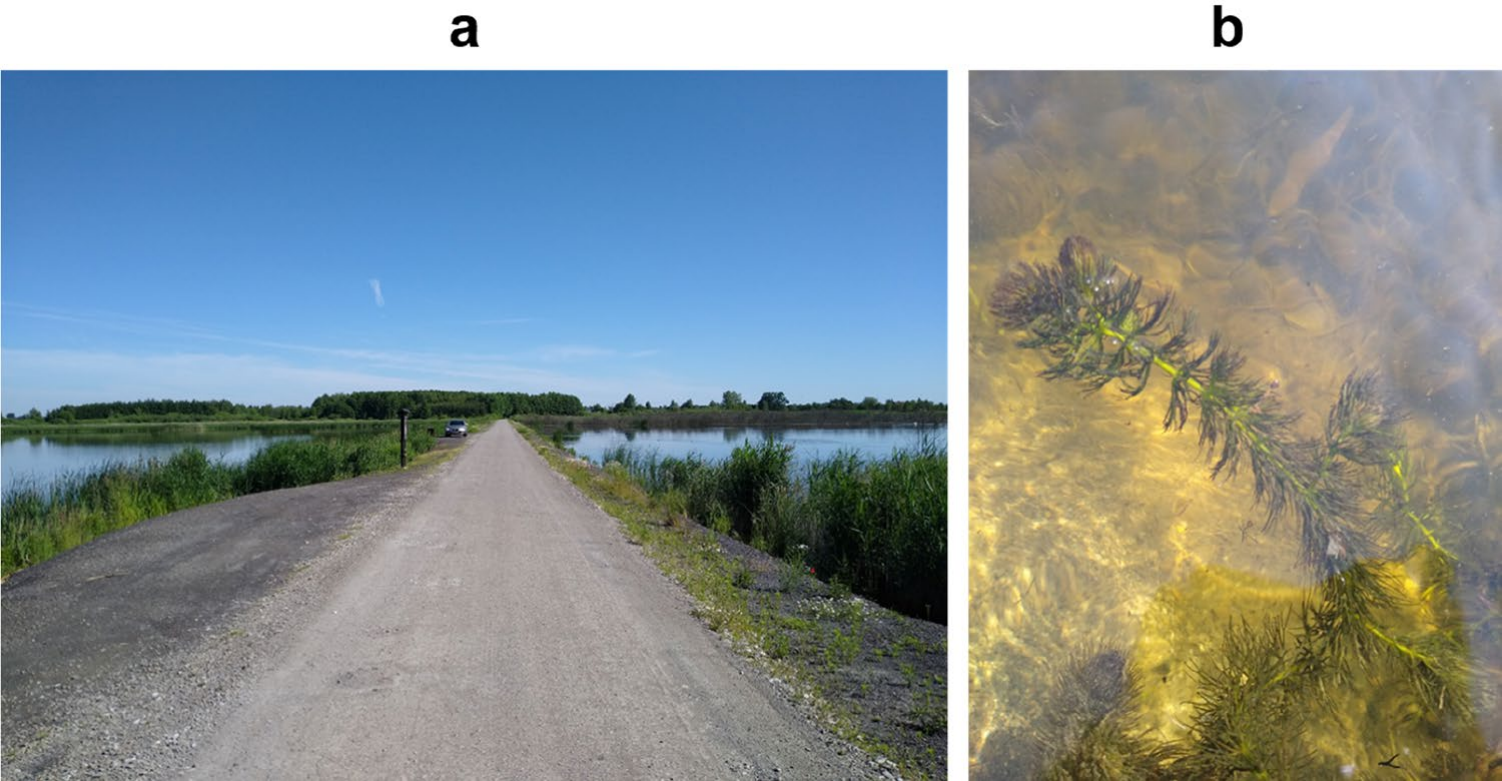
(**a**) The Nadrybie reservoir. (**b**) *Ceratophyllum demersum*.

The Nadrybie reservoir was created in 1984 as a result of land subsidence caused by underground coal mining by the "Bogdanka'' mine. As a result of its activity, an artificial basin of land was created, which, due to the shallow groundwater level, was filled with water^38^. The resulting basin, including the swamps, now covers an area of about 30 ha and is divided by a dyke made of coal gangue into two unequal parts: the larger western part (the area of open water is about 10 ha—own measurements) and the much smaller eastern part (the area of open water is about 2 ha—own measurements) (Fig. 1a).

The object of the study was the smaller eastern part of the reservoir with a maximum depth of 1.4 m, where underwater vegetation developed intensively. Therefore, this part of the reservoir was characterized by clear-water conditions with a small amount of suspended matter, which favored the development of submerged vegetation with the dominance of *C. demersum* (Fig. 1b). Intense photosynthesis resulted in high water pH values, with average pH 8.35 and 8.26 in 2019 and 2020, respectively (Table 1). In the studied reservoir, the values of electrical conductivity were typical for freshwater with low ion content (ca. 350 µS cm^−1^), and the values of chemical factors remained at a high level (Table 1).

**Table 1:** Physico-chemical and biological factors of the Nadrybie reservoir water measured during two growing seasons (April-September 2019 and 2020).

Parameter	2019		2020	
	Average	SD	Average	SD
N-NH_4_ (mg L^−1^)	0.38	0.07	0.30	0.12
N-NO_3_ (mg L^−1^)	6.48	0.74	3.60	0.86
TN (mg L^−1^)	8.18	0.61	5.44	1.10
TSI (TN)	85	1	79	3
P-PO_4_ (mg L^−1^)	0.16	0.06	0.21	0.05
TP (mg L^−1^)	0.25	0.06	0.32	0.08
TSI (TP)	83	4	87	4
pH	8.35	0.35	8.26	0.42
Electrical conductivity (µS cm^−1^)	351.17	58.18	334.00	52.12
Water temp. (°C)	19.15	5.58	17.32	5.62
CHL-a (µg L^−1^)	20.50	9.15	17.16	8.98
TSI (CHL)	59	4	57	5

*SD* standard deviation.

The average values of the Carlson index based on the total nitrogen TSI (TN) and phosphorus TSI (TP) fraction were always well above 70, which suggests hypertrophy and overfertilization of the waters of this reservoir. Such high values are characteristic of waters dominated by phytoplankton. However, the studied reservoir was overgrown with the submerged macrophyte *C. demersum*, its waters were very transparent, and the euphotic zone most often reached the bottom. Therefore, the Carlson index in this case did not reflect the proper trophic state of the reservoir. Similar findings were reported by Ejankowski and Lenard^10^ in a natural lake with macrophyte domination surrounded by an earth dyke and cut off catchment runoff. Noticeable is also the comparison of the concentration of the dissolved and total fractions of nitrogen and phosphorus. The dissolved nitrogen fractions available to plants (N–NH_4_ and N–NO_3_) accounted for over 70% of the total nitrogen fraction (TN), while the dissolved phosphorus fraction (P–PO_4_) accounted for over 60% of the total phosphorus fraction (TP, Table 1). Thus, these values confirmed the high availability of nutrients in the water, which were not bound due to the lack of massive phytoplankton blooms. This is consistent with the previous results obtained by Mjelde and Faafeng^13^ in small, shallow and macrophyte-dominated Norwegian lakes. The average concentration of chlorophyll-*a* in the Nadrybie reservoir was 20.50 µg L^−1^ in 2019 and 17.16 µg L^−1^ in 2020, and indicated moderate development of phytoplankton. This was also confirmed by the values of the Carlson index based on the chlorophyll-*a* values TSI (CHL), which classified the Nadrybie reservoir as slightly eutrophic waters. This lack of agreement in the trophic state estimation between the values of TSI (TN, TP, and CHL) may provide additional insight into the reservoir dynamics, as already pointed out by Carlson and Havens^39^. A consequence of the hypothetical phytoplankton bloom could be an increase in water turbidity and deterioration of light conditions for the development of aquatic macrophytes. However, this was not the case of the Nadrybie reservoir, and the intensive development of underwater vegetation dominated by *C. demersum* was possible. This species is known for its allelopathic properties, i.e. inhibition of the development of phytoplankton, the second group of primary producers in the aquatic environment competing for the same environmental resources (biogenic compounds, light necessary for photosynthesis). The inhibiting effect of *C. demersum* was often found against epiphytic diatoms, planktonic green algae, and chroococcal and filamentous cyanobacteria^11,20–23,40–42^. However, there were also some reports of a stimulating effect of *C. demersum* on other algae^25–28^. Therefore, this species can be regarded as a stabilizer of clear-water status in shallow water bodies^12,13^. This relationship between the aquatic macrophytes and phytoplankton in the Nadrybie reservoir resulted in the intensive development of *C. demersum*, and the limited development of phytoplankton contributed to the small amount of abioseston or bioseston suspension covering the plants, which greatly facilitated the collection and preparation of the plants for further research.

### Extraction and content analysis

The total amount of phenolic compounds (TPC) in the *C. demersum* dry ethanolic extract (DE) was found to be 18.50 ± 0.02 mg gallic acid equivalent/g, with flavonoids constituting the predominant part (16.09 ± 0.28 mg quercetin equivalent/g DE). The TPC value was similar to that reported by Li et al.^43^. However, it was lower than the value determined by Emsen and Dogan^44^ and Murat et al.^45^ in extracts prepared from plant material collected in Turkey or propagated in vitro (64.73 ± 1.16 and 528.26 ± 4.07 mg/g extract, respectively)^44,45^. Kartal et al. also reported a higher extract yield (16.5%). On the other hand, we observed a several times higher level of flavonoids than that shown by Emsen & Dogan (2.63 ± 0.34 μg/mg). The differences may largely result from geographical or environmental factors (e.g. differences in the surface water temperature, intensity of solar radiation)^46^.

The LC–MS analysis revealed the presence of a variety of polyphenols in the *C. demersum* extract (Table 2; Supplementary Figs. S1 and S2 in the Supplementary Information). This aquatic macrophyte was found to contain eight phenolic acids, including benzoic (gallic, protocatechuic, 4-hydroxybenzoic, and syringic) and cinnamic (5-caffeoylquinic, *p*-coumaric, ferulic, and isoferulic) acid derivatives. Syringic acid (a molecule with proven pleiotropic activity)^46^ was the prevailing compound (1276.67 ± 35.36 µg/g dry extract), followed by 4-hydroxycinnamic acid (543.00 ± 11.31 µg/g DE). A significant amount of *p*-coumaric and protocatechuic acid was detected as well (108.85 and 93.18 µg/g DE, respectively). A relatively low concentration of 5-caffeoylquinic acid was determined. The other detected phenolic acids were present only in trace amounts (Table 2).

**Table 2:** LC–ESI–MS/MS analysis of the *C. demersum* dry extract.

Compound	µg/g of the dry extract	± SD
Phenolic acids		
Gallic	BQL	
Protocatechuic	93.18	3.36
5-caffeoylquinic acid	1.48	0.04
4-hydroxybenzoic	543.00	11.31
Syringic	1276.67	35.36
p-coumaric	108.85	5.06
Ferulic	BQL	
Isoferulic	BQL	
Flavonoid aglycones		
Catechin	7.47	0.07
Taxifolin	15.23	0.40
Luteolin	209.83	2.83
Eriodictyol	3.67	0.05
Quercetin	47.18	0.85
Apigenin	4286.25	45.96
Isorhamnetin	5.46	0.21
Sakuranetin	BQL	
Flavonoid glycosides		
Luteolin-3,7-diglucoside	746.50	32.92
Eriodictyl-7-O-rutinoside	BQL	
Rutin	53.40	1.66
Hyperoside	303.33	3.18
Luteolin-7-glucoside	2657.50	109.43
Isoquercetin	1022.50	23.98
Nicotiflorin	133.25	7.41
Narcissoside	553.33	40.93
Isorhamnetin-3-O-glucoside	177.75	5.50
Quercitrin	40.25	0.21
Apigenin-7-O-glucoside	6862.50	180.28
Naringenin-7-O-glucoside	52.88	2.31

*BQL* below the quantification limit.

Most of the detected phenolic acids (ferulic, p-coumaric, gallic, protocatechuic, 4-hydroxybenzoic, and syringic) were previously identified in *C. demersum*^45,47,48^. However, the presence of 5-caffeoylquinic and isoferulic acid has not been reported in this plant material to date.

Eight flavonoid aglycones and twelve flavonoid glycosides were found in *C. demersum*. The extract contained large quantities of apigenin and its 7-*O*-glucoside (4286.25 and 6862.50 µg/g DE, respectively). This finding is in agreement with previous studies showing the presence of apigenin with its glycosides in the species^47,49^. These compounds have been gaining increasing interest recently due to their anti-cancer potential^50,51^. Luteolin with its two glycosides (luteolin 3′,7′-diglucoside and luteolin-7-*O*-glucoside) accounted for another large part of the *C. demersum* extract (in total 3613.83 µg/g DE), followed by quercetin and its derivatives (rutin, hyperoside, isoquercetin, quercitrin; in total 1466.67 µg/g DE). These groups of secondary metabolites can also exert pharmacological activity by affecting several biochemical targets^52,53^.

Our study has revealed for the first time the presence of isorhamnetin with its two glycosides (narcissoside and isorhamnetin-3-*O*-glucoside), sakuranetin (naringenin 7-*O*-methyl ether), taxifolin, and eriodictyol (with its 7-*O*-glucoside) in the studied macrophyte. We have also identified and quantified catechin, kaempferol-3-*O*-rutinoside, and naringenin 7-*O*-glucoside in the *C. demersum* extract.

To the best of our knowledge, this is the first phytochemical study of *C. demersum* collected in Poland. Its ethanolic extract was found to contain several chemical structural classes of polyphenols. It can be noticed that the secondary metabolism of the species is similar to that of terrestrial higher plants. The majority of the detected compounds possess proven biological potential, e.g. chemopreventive, anti-inflammatory, immunoregulatory, antidiabetic, and antiangiogenic activity, and can act as modulators of cell-signaling pathways^50–52,54–56^. Therefore, *C. demersum* may be considered a valuable source of polyphenols for potential health-beneficial or medicinal applications and certainly deserves more scientific interest.

### Antiproliferative activity assay

Biological tests were conducted to verify the anticancer activity of the tested extract and thus confirm its potential usefulness in supporting cancer prevention. For this purpose, in vitro cell-based assays were used. In order to measure cellular metabolic activity, the MTT assay was implemented as an indicator of cell viability, proliferation, and cytotoxicity.

**Figure 2:**
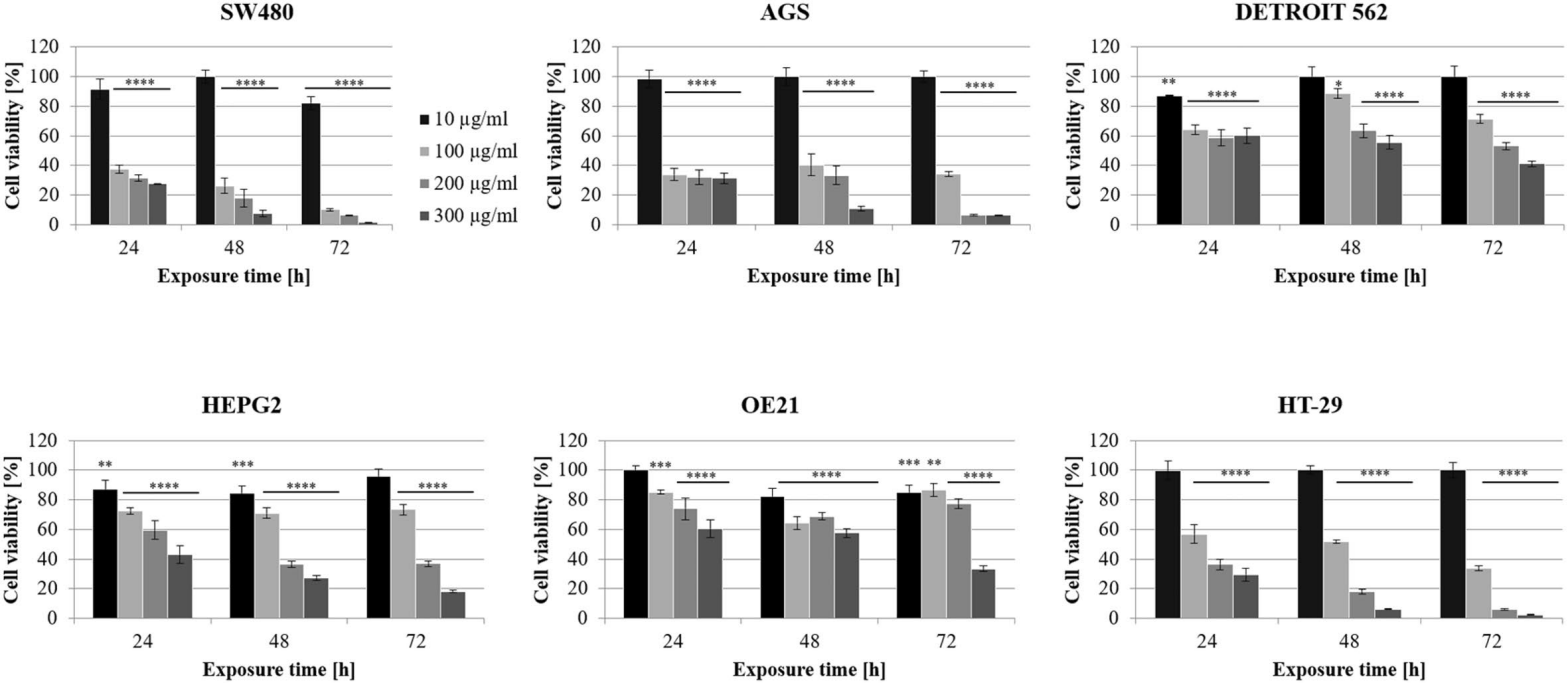
Effect of *C. demersum* crude extract on the viability of different human tumor cell lines at the concentrations of 10–300 µg/ml after 24, 48, and 72 h of incubation, assessed with the use of the MTT assay. The results are presented as the mean ± SD. ANOVA post hoc Dunnett’s test: *p < 0.05, **p < 0.01, ***p < 0.001, ****p < 0.0001 versus control, n = 3.

The studies showed a potent effect of the extract on the viability of tumor cells in a dose and time-dependent manner (Fig. 2). It was found that the HT-29, SW480 (colorectal adenocarcinoma), and AGS (gastric adenocarcinoma) cell lines were particularly sensitive, as their viability reached a value below 10% in the case of 200 and 300 µg/ml after the 72-h treatment. Nevertheless, in the case of the less sensitive cell lines, a 58.97, 66.52, and 81.78% decrease in the viability of the Detroit 562, OE21, and Hep G2 cells, respectively, was observed after the 72-h treatment (300 µg/ml), which can be regarded as high activity, considering the natural origin of the tested compound. For comparison, the ethanolic extract of *C. demersum* collected in Kolhapur, Maharashtra (India) obtained and tested by Awati et al. showed lower cytotoxic activity against the HT-29 cell line, reaching an IC_50_ value of 571.3 µg/ml after 48 h of incubation^57^. Our results prove the significant potential of *C. demersum* as a source of anticancer substances that can be used as an element of the diet in the prevention of gastrointestinal cancers. According to World Cancer Research Fund International, 18.1 million cancer cases were diagnosed in 2020 worldwide. Among these cases, colorectal cancer accounted for 10%. To fight with these disorders, different treatments, i.e., chemotherapy, radiotherapy, or surgery, are used. However, all of them suffer from different serious drawbacks^58^ such as strong side effects and toxicities^59^. Thus, new strategies are needed. An approach that can be really applied is the preventive action. Therefore, the results obtained here and numerous studies on natural compounds may be the starting point for new therapies.

### Annexin V/propidium iodide apoptosis study

**Figure 3:**
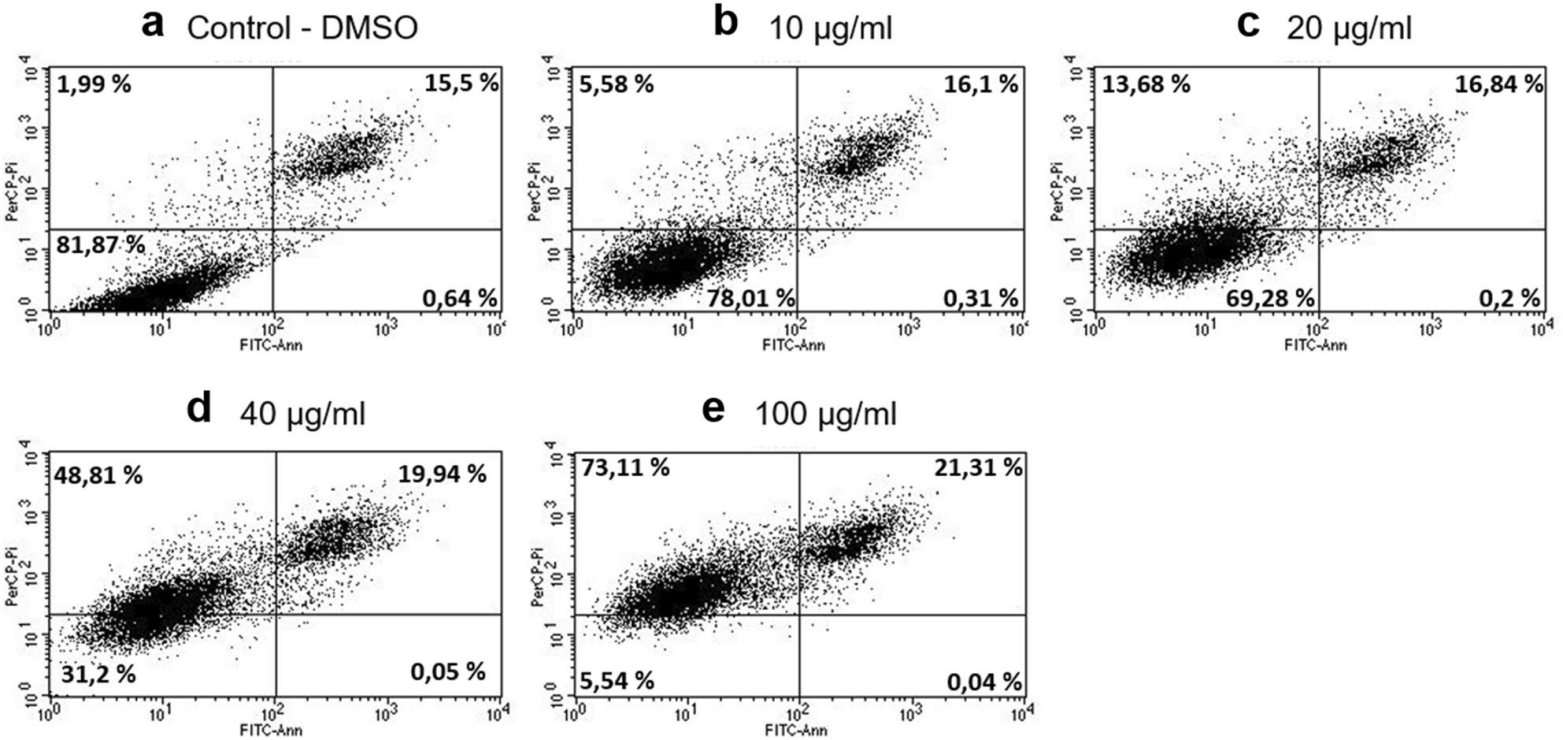
Effects of *C. demersum* crude ethanolic extract on SW480 cells measured with the annexin-V/propidium iodide (PI) assay. (**a**) Untreated control cells; (**b**–**e**) cells treated with 10 μg/ml, 20 μg/ml, 40 μg/ml, and 100 μg/ml of *C. demersum* crude extract for 24 h. Live cells (annexin-V-negative and PI negative) are shown in the lower left quadrant, early apoptotic cells (annexin-V-positive and PI-negative) in the lower right quadrant, late apoptotic in the upper right quadrant, and necrotic cells (annexin-V-positive and PI-positive) in the upper left quadrant.

For elucidation of the mode of action of the tested extracts, the flow cytometry technique was adopted to show the mechanism of the average activity of active molecules that are components of the extract. The SW480 cell line was selected for the flow cytometric analysis as the most sensitive line indicated by the MTT test. The tested crude extract caused a concentration-dependent increase in late apoptosis and necrosis in the SW480 cells. The treatment of the SW480 cells with 10, 20, 40, and 100 μg/ml for 24 h increased the percentage of late apoptotic cells from 16.1 to 21.31% in a concentration-dependent manner, compared to the untreated control cells (Fig. 3). Furthermore, a significant increase in the necrosis rate from 5.58% at 10 μg/ml, 13.68% at 20 μg/ml, and 48.81% at 40 μg/ml to 73.11% at 100 μg/ml was found.

### Ecotoxicity and safety studies on a zebrafish model

**Figure 4:**
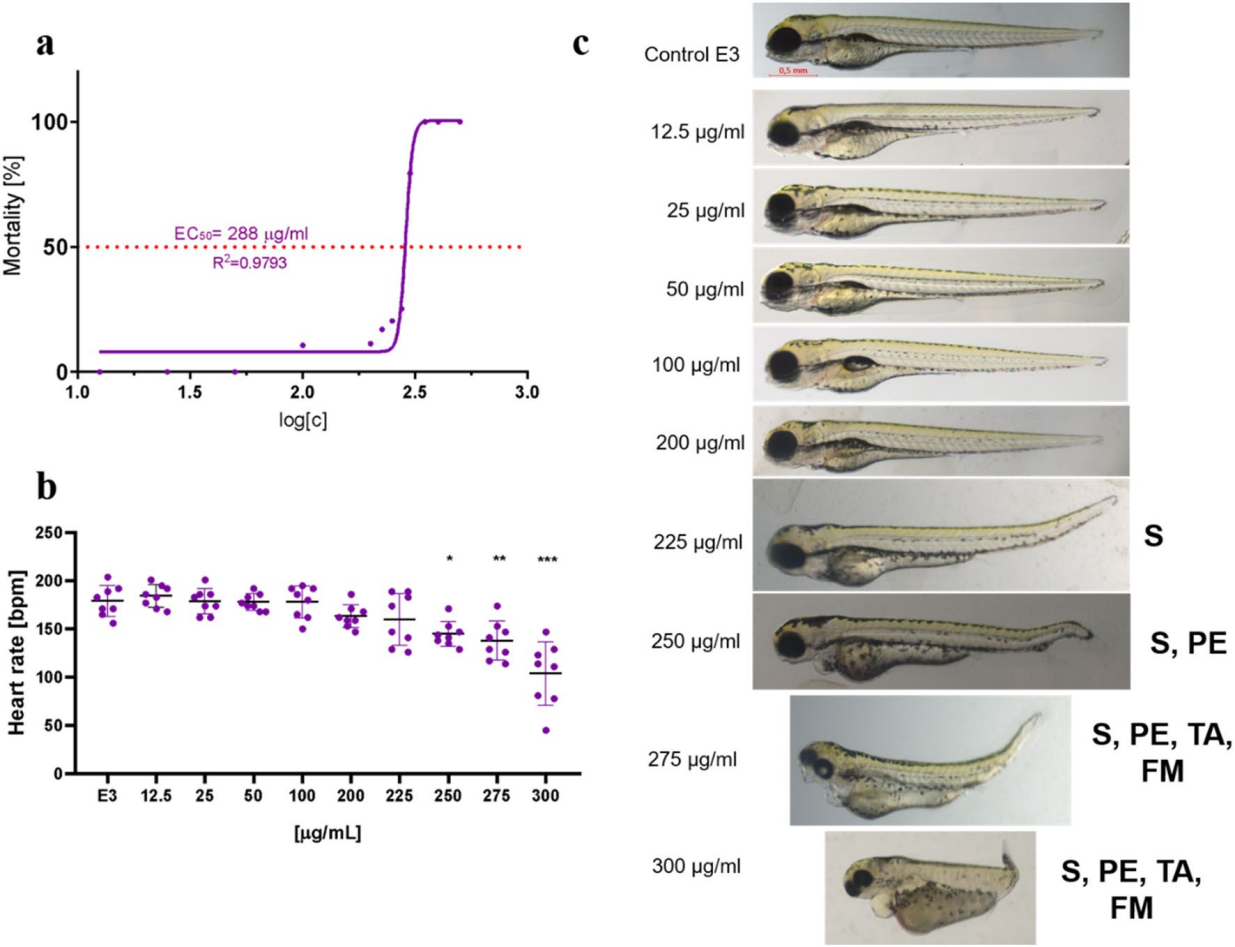
(**a**) Mortality level, (**b**) heart rate, (**c**) toxicity and teratogenicity in *Danio rerio* zebrafish embryos exposed to the *C. demersum* ethanolic extract: S, scoliosis; PE, pericardial edema; TA, tail autophagy; FM, face malformations. ANOVA post hoc Tukey test: *p < 0.05, **p < 0.01, ***p < 0.001.

In order to verify the ecotoxicity of the extract towards living organisms as well as the safety of the use of the preparation, a *Danio rerio* fish model was employed. The extract was tested against fish embryos in order to determine its embryotoxic properties (Fig. 4). As shown by the results, the extract exhibited signs of toxicity at high concentrations (LC_50_ = 288 µg/ml), compared to the effective dose against the tumor cell lines. Moreover, the extract caused the first signs of developmental malformations (scoliosis) at the concentration of 225 µg/ml. The wider spectrum of malformations (scoliosis, pericardial edema) appeared at the dose of 250 µg/ml, and further tail autophagy as well as face malformations were observed at the highest concentrations (275–300 µg/ml). Significant changes in the heart rate were noted at the dose of 250 µg/ml. The safety study of the *C. demersum* extract conducted using the *D. rerio* model revealed that the preparation is safe to animals up to the concentration of 225 µg/ml, while lower doses in the range of 10–200 µg/ml are effective against cancer cells (Figs. 2, 3).

## Conclusion

Cancer chemoprevention is a good strategy to mitigate the problems associated with tumor incidence. It consists in the use of natural and synthetic agents to suppress, prevent, or delay tumorigenesis by disturbing early stages of carcinogenesis. Clinical studies have shown the effectiveness of this approach in managing cancers of different origins. Curcumin, resveratrol, emodin, quercetin, genistein, and epigallocatechin gallate are examples of such phytochemicals showing clinical relevance. An advantage of chemopreventive agents over chemotherapeutics is the ability of regulation of multiple survival pathways without induction of toxicity^60^.

Plants, including these of water origin, are the main source of chemopreventive agents. Thus, our studies fit in the trend to explore the water environment in search of novel sources of anticancer substances. We suggest that aquatic plants should be acquired for research from macrophyte reservoirs exhibiting a small amount of suspension. The *Ceratophyllum demersum* plants obtained from such an anthropogenic reservoir appeared to be a rich source of biologically active phenolic acids and flavonoids, whose activities are widely reported. In our case, the set of these chemicals showed anticancer properties, with special emphasis on cancer cells derived from the gastrointestinal tract. The ecotoxicity/embryotoxicity studies using *Danio rerio* revealed the safety of the crude extract from *Ceratophyllum demersum*, which may be considered as a future chemopreventive agent against gastrointestinal tract cancers.

### Supplementary Information

Supplementary Information.

## Data Availability

All data generated or analyzed during this study are included in this paper. Any additional data or subset of datasets used for this study are available from the corresponding author upon request.

## References

[1] Lojek A, Denev P, Ciz M, Vasicek O, Kratchanova M (2014). The effects of biologically active substances in medicinal plants on the metabolic activity of neutrophils. Phytochem. Rev..

[2] Wink, M., Krauss, G. & Nies, D. Evolution of secondary metabolism in plants. *Ecol. Biochem. Environ. Interspec. Interact.* 39–47 (2015).

[3] Azmir J (2013). Techniques for extraction of bioactive compounds from plant materials: A review. J. Food Eng..

[4] Rubio L, Motilva M, Romero M (2013). Recent advances in biologically active compounds in herbs and spices: A review of the most effective antioxidant and anti-inflammatory active principles. Crit. Rev. Food Sci. Nutr..

[5] van Wyk, B. E. & Wink, M. *Medicinal Plants of the World*. (Timber Press, 2017).

[6] Espinosa-Leal C, Puente-Garza C, Garcia-Lara S (2018). In vitro plant tissue culture: Means for production of biological active compounds. Planta.

[7] Hostettmann K, Wolfender J, Terreaux C (2001). Modern screening techniques for plant extracts. Pharm. Biol..

[8] Huie C (2002). A review of modern sample-preparation techniques for the extraction and analysis of medicinal plants. Anal. Bioanal. Chem..

[9] Scheffer M, Jeppesen E, Sondergaard M, Christoffersen K (1998). Alternative stable states. Struct. Role Subbmerg. Macrophytes Lakes.

[10] Ejankowski W, Lenard T (2014). Trophic state of a shallow lake with reduced inflow of surface water. Arch. Environ. Prot..

[11] Gross, E., Erhard, D. & Ivanyi, E. Allelopathic activity of Ceratophyllum demersum L. and Najas marina ssp intermedia (Wolfgang) Casper. *Hydrobiologia* **506**, 583–589. 10.1023/B:HYDR.0000008539.32622.91 (2003).

[12] Hilt S, Gross E (2008). Can allelopathically active submerged macrophytes stabilise clear-water states in shallow lakes?. Basic Appl. Ecol..

[13] Mjelde M, Faafeng B (1997). Ceratophyllum demersum hampers phytoplankton development in some small Norwegian lakes over a wide range of phosphorus concentrations and geographical latitude. Freshw. Biol..

[14] Dokulil M (2010). The impact of climate change on lakes in Central Europe. Impact Clim. Change Eur. Lakes.

[15] Scheffer M, van Nes E (2007). Shallow lakes theory revisited: Various alternative regimes driven by climate, nutrients, depth and lake size. Hydrobiologia.

[16] Hultén, E. & Fries, M. *Atlas of North European vascular plants. North of the Tropic of Cancer*. (Koeltz Scientific Books, 1986).

[17] Ejankowski W, Lenard T (2016). The effect of ice phenology exerted on submerged macrophytes through physicochemical parameters and the phytoplankton abundance. J. Limnol..

[18] Ejankowski, W. & Solis, M. Response of hornwort (Ceratophyllum demersum L.) to water level drawdown in a turbid water reservoir. *Appl. Ecol. Environ. Res.* **13**, 219–228 (2015).

[19] Ahluwalia, A. S. in *Allelopathy: Current Trends and Future Applications* (eds Z A Cheema, M Farooq, & A Wahid) 485–509 (Springer-Verlag, 2013).

[20] Hu H, Hong Y (2008). Algal-bloom control by allelopathy of aquatic macrophytes—A review. Front. Environ. Sci. Eng. China.

[21] Jasser I (1995). The influence of macrophytes on a phytoplankton community in experimental conditions. Hydrobiologia.

[22] Kogan, S. I. & Chinnova, G. A. Relations between Ceratophyllum demersum (L.) and some blue-green algae. *Hydrobiol. J.* **8**, 14–19 (1972).

[23] Mohamed Z (2017). Macrophytes-cyanobacteria allelopathic interactions and their implications for water resources management-A review. Limnologica.

[24] Nakai S, Inoue Y, Hosomi M, Murakami A (1999). Growth inhibition of blue-green algae by allelopathic effects of macrophyte. Water Sci. Technol..

[25] Dong J, Lu J, Li G, Song L (2013). Influences of a submerged macrophyte on colony formation and growth of a green alga. Aquat. Biol..

[26] Dong J (2018). Colony formation by the green alga Chlorella vulgaris in response to the competitor Ceratophyllum demersum. Hydrobiologia.

[27] Mulderij G, Van Donk E, Roelofs J (2003). Differential sensitivity of green algae to allelopathic substances from Chara. Hydrobiologia.

[28] van Donk E, van de Bund W (2002). Impact of submerged macrophytes including charophytes on phyto- and zooplankton communities: Allelopathy versus other mechanisms. Aquat. Bot..

[29] Kleiven S, Szczepanska W (1988). The effects of extracts from chara-tomentosa and 2 other aquatic macrophytes on seed-germination. Aquat. Bot..

[30] Viveros-Legorreta JL, Sarma SS, Guerrero-Zúñiga LA, Rodríguez-Dorantes A (2016). Effect of the nature of Ceratophyllum demersum extracts on Lactuca sativa seedlings. Int. J. Curr. Res. Biosci. Plant Biol..

[31] Nusch EA (1980). Comparison of different methods for chlorophyll and phaeopigment determination. Arch. Hydrobiol. Beih. Ergebn. Limnol..

[32] Dojlido, J. & Hermanowicz, W. *Physical–chemical investigation of water and sewage*. (Arkady, 1999).

[33] Carlson R (1977). Trophic state index for lakes. Limnol. Oceanogr..

[34] Kratzer C, Brezonik P (1981). A carlson-type trophic state index for nitrogen in florida lakes. Water Resour. Bull..

[35] Matuszkiewicz W (2001). Guide for identification of the plant communities of Poland.

[36] Olech, M., Łyko, L. & Nowak, R. Influence of accelerated solvent extraction conditions on the LC-ESI-MS/MS polyphenolic profile, triterpenoid content, and antioxidant and anti-lipoxygenase activity of. *Antioxidants (Basel)* **9**. 10.3390/antiox9090822 (2020). 10.3390/antiox9090822 PMC7555744 32899188

[37] Olech, M., Pietrzak, W. & Nowak, R. Characterization of free and bound phenolic acids and flavonoid aglycones. *Molecules* **25**. 10.3390/molecules25081804 (2020). 10.3390/molecules25081804 PMC7221549 32326454

[38] Radwan, S. *et al.* Ecological characteristics of the depression reservoirs—preliminary results. *Int. Assoc. Theor. Appl Limnol. 28*, 178–182 (2002).

[39] Carlson R, Havens K (2005). Simple graphical methods for the interpretation of relationships between trophic state variables. Lake Reservoir Manag..

[40] van Donk E, Gulati RD (1995). Transition of a lake to turbid state 6 years after biomanipulation: Mechanisms and pathways. Water Sci. Technol..

[41] Lupoae, M., Lupoae, P., Borda, D., Cristea, V. & Bocioc, E. Allelopathic potential of the Ranunculus rionii lagger and Ceratophyllum demersum L. extracts against microbial and microalgal cultures. *Environ. Eng. Manag. J.* **15**, 473–480 (2016).

[42] Kurashov, E., Fedorova, E., Krylova, J. & Mitrukova, G. Assessment of the potential biological activity of low molecular weight metabolites of freshwater macrophytes with QSAR. *Scientifica* **2016**. 10.1155/2016/1205680 (2016). 10.1155/2016/1205680 PMC4854990 27200207

[43] Li, Z., Tu, Z., Wang, H. & Zhang, L. Ultrasound-assisted extraction optimization of α-glucosidase inhibitors from. *Molecules* **25**. 10.3390/molecules25194507 (2020). 10.3390/molecules25194507 PMC7582508 33019644

[44] Emsen, B. & Dogan, M. Evaluation of antioxidant activity of *in vitro* propagated medicinal Ceratophyllum demersum L. extracts. *Acta Sci. Pol. Hort. Cultus* **17**, 23–33 (2019).

[45] Kartal M (2009). Antioxidant and anticholinesterase assets and liquid chromatography-mass spectrometry preface of various fresh-water and marine macroalgae. Pharmacogn. Mag..

[46] Escobar-Bravo R, Klinkhamer PG, Leiss KA (2017). Interactive effects of UV-B light with abiotic factors on plant growth and chemistry, and their consequences for defense against arthropod herbivores. Front. Plant Sci..

[47] Li, Z., Tu, Z., Wang, H. & Zhang, L. Ultrasound-assisted extraction optimization of α-glucosidase inhibitors from Ceratophyllum demersum L. and identification of phytochemical profiling by HPLC-QTOF-MS/MS. *Molecules* **25**, 1. 10.3390/molecules25194507 (2020). 10.3390/molecules25194507 PMC7582508 33019644

[48] Usenko O, Konovets I (2014). Analysis of pheolcarbonic acids content in phytomass of higher aquatic plants. Hydrobiol. J..

[49] Bankova V, Ivanova P, Christov R, Popov S, Dimitrova-Konaklieva ST (1995). Secondary metabolites of ceratophyllum demersum. Hydrobiologia.

[50] Javed Z (2021). Apigenin role as cell-signaling pathways modulator: Implications in cancer prevention and treatment. Cancer Cell Int..

[51] Liu MM (2020). Apigenin 7-O-glucoside promotes cell apoptosis through the PTEN/PI3K/AKT pathway and inhibits cell migration in cervical cancer HeLa cells. Food Chem. Toxicol..

[52] De Stefano A (2021). Anti-inflammatory and proliferative properties of luteolin-7-o-glucoside. Int. J. Mol. Sci..

[53] Hirpara KV, Aggarwal P, Mukherjee AJ, Joshi N, Burman AC (2009). Quercetin and its derivatives: synthesis, pharmacological uses with special emphasis on anti-tumor properties and prodrug with enhanced bio-availability. Anticancer Agents Med. Chem..

[54] Jantan I (2021). Dietary polyphenols suppress chronic inflammation by modulation of multiple inflammation-associated cell signaling pathways. J. Nutr. Biochem..

[55] Nowak, R., Olech, M. & Nowacka-Jechalke, N. in *Polyphenols in human health and disease* (eds Ronald Ross Watson, Victor Preedy, & Sherma Zibadi) 1289–1305 (Academic Press, 2013).

[56] Srinivasulu C, Ramgopal M, Ramanjaneyulu G, Anuradha CM, Suresh Kumar C (2018). Syringic acid (SA)—a review of its occurrence, biosynthesis, pharmacological and industrial importance. Biomed. Pharmacother..

[57] Awati, S. S., Singh, S. K. & Wadkar, K. A. In vitro Antioxidant potential and Anticancer activity of Ceratophyllum demersum Linn. extracts on HT-29 human colon cancer cell line. **14**, 28–36 (2021).

[58] Karpuz M, Silindir-Gunay M, Ozer AY (2018). Current and future approaches for effective cancer imaging and treatment. Cancer Biother. Radiopharm..

[59] Nobili S (2009). Natural compounds for cancer treatment and prevention. Pharmacol. Res..

[60] Shankar MG, Swetha M, Keerthana CK, Rayginia TP, Anto RJ (2021). Cancer chemoprevention: A strategic approach using phytochemicals. Front. Pharmacol..

