# Supplementary material for: Ceratophyllum demersum the submerged macrophyte from the mining subsidence reservoir Nadrybie Poland as a source of anticancer agents
Source: Sci Rep. 2024 Mar 20;14:6661. doi: 10.1038/s41598-024-57375-6 (PMC10954700; doi:10.1038/s41598-024-57375-6)
Supplement: Supplementary file 1 — Supplementary Information. [file 41598_2024_57375_MOESM1_ESM.docx]

**Plants of mining subsidence reservoir as a source of anticancer agents. The case of submerged macrophyte *Ceratophyllum demersum***

**Maciej Masłyk ^a^, Tomasz Lenard ^b^, Marta Olech ^c^, Aleksandra Martyna ^a^, Małgorzata Poniewozik ^d^, Anna Boguszewska-Czubara^e^ , Elżbieta Kochanowicz ^a^, Paweł Czubak ^a^, and Konrad Kubiński ^a *^**

^a^ Department of Molecular Biology, The John Paul II Catholic University of Lublin, ul. Konstantynów 1i, 20-708 Lublin, Poland

^b^ Department of Animal Physiology and Toxicology, The John Paul II Catholic University of Lublin, ul. Konstantynów 1i, 20-708 Lublin, Poland

^c^ Department of Pharmaceutical Botany, Medical University of Lublin, ul. Chodźki 1, 20-093 Lublin, Poland

^d^ Department of Plant Physiology and Biotechnology, The John Paul II Catholic University of Lublin, ul. Konstantynów 1i, 20-708 Lublin, Poland

^e^ Chair and Department of Medical Chemistry, Medical University of Lublin, ul. Chodźki 4a, 20-093 Lublin, Poland

* Correspondence: kubin@kul.pl

**SUPPLEMENTARY INFORMATION**

**Supplementary Table S1.** Optimized parameters for the LC-ESI-MS/MS qualitative analysis.

| **Compound** | **t_R_ [min]** | **[M-H]^-^**  **[m/z]** | **Fragment ions**  **[m/z]** | **Colision energy [eV]** |
| --- | --- | --- | --- | --- |
| **Phenolic acids** | | | | |
| Gallic acid | 5.16 | 168.7 | 78.9  124.9 | -36  -14 |
| Protocatechuic acid | 8.42 | 152.9 | 80.9  107.8 | -26  -38 |
| 5-Caffeoylquinic acid | 9.29 | 352.9 | 191.1  178.9 | -30  -30 |
| 4-Hydroxybenzoic acid | 10.84 | 136.8 | 93 | -18 |
| Gentisic acid | 11.45 | 152.9 | 80.9  107.8 | -110  -52 |
| Vanilic acid | 11.49 | 166.8 | 107.9  123 | -18  -12 |
| Syringic acid | 11.44 | 196.9 | 122.8  181.9 | -24  -12 |
| Caffeic acid | 11.69 | 178.7 | 88.9  134.9 | -30  -30 |
| 3-Hydroxybenzoic acid | 12.10 | 136.9 | 93  75 | -16  -48 |
| 4-Hydroxycinnamic acid  (*p*-coumaric acid) | 14.10 | 162.7 | 119  93 | -14  -44 |
| Sinapic acid | 14.47  14.94 | 222.8 | 121  148.9 | -36  -20 |
| Ferulic acid | 14.87 | 192.8 | 133.9  177.9 | -16  -12 |
| Isoferulic acid | 10.55 | 192.8 | 133.9  177.9 | -16  -12 |
| Rosmarinic acid | 15.91 | 358.7 | 160.8  196.8 | -20  -22 |
| 3-Hydroxycinnamic acid  (*m*-Coumaric acid) | 16.05 | 162.8 | 119  91 | -14  -36 |
| 2-Hydroxycinnamic acid  (*o*-Coumaric acid) | 17.15 | 162.8 | 119  91 | -14  -36 |
| Salicylic acid | 17.91 | 136.8 | 93  75 | -16  -48 |
| **Flavonoid aglycones** | | | | |
| Catechin | 9.64 | 288.8 | 244.9  109 | -16  -32 |
| Naringenin | 14.52 | 270.8 | 119  150.9 | -34  -22 |
| Taxifolin | 15.15 | 302.7 | 124.9  284.8 | -26  -14 |
| Myricetin | 16.57 | 316.7 | 136.9  150.9 | -32  -26 |
| Luteolin | 17.82 | 284.7 | 132.9  150.9 | -38  -26 |
| Eriodictyol | 17.89 | 286.7 | 134.9  150.9 | -32  -18 |
| Quercetin | 17.94 | 300.7 | 150.9  178.8 | -26  -20 |
| 3-*O*-Methylquercetin | 18.11 | 314.7 | 299.8  270.8 | -18  -26 |
| Apigenin | 18.64 | 268.8 | 117  106.8 | -44  -34 |
| Kaempferol | 18.85 | 284.7 | 116.8  93 | -46  -52 |
| Isorhamnetin | 18.99 | 314.7 | 299.7  150.9 | -20  -30 |
| Rhamnetin | 20.10 | 314.7 | 165  120.9 | -24  -36 |
| Sakuranetin | 21.67 | 284.7 | 118.9  164.8 | -34  -20 |
| Prunetin | 21.98 | 282.8 | 267.7  238.7 | -20  -26 |
| Rhamnazin | 22.37 | 328.7 | 270.8  313.8 | -26  -14 |
| **Flavonoid glycosides** | | | | |
| Luteolin 3’,7’-diglucoside | 11.28 | 609.1 | 285  447 | -50  -32 |
| Eriodictyol-7-*O*-rutinoside (Eriocitrin) | 11.93 | 594.8 | 286.9  150.9 | -34  -46 |
| Rutin  (Quercetin 3-*O*-rutinoside) | 11.99 | 608.7 | 299.6  270.9 | -46  -60 |
| Hyperoside  (Quercetin 3-*O*-galactoside) | 12.80 | 462.7 | 299.7  254.7 | -28  -42 |
| Luteolin-7-*O*-glucoside | 12.87 | 446.8 | 284.8  132.9 | -30  -78 |
| Isoquercetin | 13.00 | 462.7 | 299.7  270.7 | -30  -44 |
| Kaempferol – 3-*O*-rutinoside (Nicotiflorin) | 13.31 | 592.7 | 284.8  226.7 | -38  -68 |
| Isorhamnetin-3-*O*-rutinoside (Narcissoside) | 13.52 | 622.8 | 314.9  298.8 | -40  -52 |
| Astragalin | 14.66 | 446.7 | 226.8  254.8 | -54  -40 |
| Isorhamnetin-3-*O*-glucoside | 14.76 | 476.8 | 313.9  270.9 | -30  -44 |
| Quercitrin | 14.83 | 446.7 | 299.7  270.7 | -30  -40 |
| Apigenin 7-*O*-glucoside | 14.91 | 430.7 | 267.7  116.9 | -38  -84 |
| Naringenin 7-*O*-glucoside | 15.12 | 432.7 | 270.8  118.9 | -22  -64 |
| Tiliroside | 17.39 | 592.8 | 284.8  254.7 | -38  -56 |

**Supplementary Table S2.** Analytical parameters used in LC-MS/MS quantitative method for compounds identified in *Ceratophyllum demersum* L. extract.

| **Compound** | **LOD**  **[ng/mL]** | **LOQ**  **[ng/mL]** | **R^2^** | **Linearity range**  **[ng/mL]** |
| --- | --- | --- | --- | --- |
| **Phenolic acids** | | | | |
| Gallic acid | 500 | 750 | 0.9989 | 750-15000 |
| Protocatechuic acid | 300 | 500 | 0.9988 | 1000-20000 |
| 5-Caffeoylquinic acid | 75 | 175 | 0.9985 | 175-5250 |
| 4-Hydroxybenzoic acid | 200 | 400 | 0.9993 | 500-10000 |
| Syringic acid | 500 | 750 | 0.9991 | 750-17500 |
| 4-Hydroxycinnamic acid  (*p*-coumaric acid) | 20 | 50 | 0.9995 | 100-3500 |
| Ferulic acid | 30 | 75 | 0.9994 | 75-2250 |
| Isoferulic acid | 25 | 60 | 0.9991 | 60-1800 |
| **Flavonoid aglycones** | | | | |
| Catechin | 25 | 50 | 0.9987 | 250-20000 |
| Taxifolin | 20 | 50 | 0.9986 | 50-5000 |
| Luteolin | 20 | 40 | 0.9980 | 40-4000 |
| Eriodictyol | 5 | 15 | 0.9982 | 15-5000 |
| Quercetin | 2 | 3 | 0.9985 | 30-3000 |
| Apigenin | 3 | 4 | 0.9987 | 12-6000 |
| Isorhamnetin | 300 | 450 | 0.9995 | 450-9000 |
| Sakuranetin | 70 | 140 | 0.9990 | 140-4500 |
| **Flavonoid glycosides** | | | | |
| Luteolin 3’,7’-diglucoside | 250 | 500 | 0.9989 | 750-15000 |
| Eriodictyol-7-*O*-rutinoside (Eriocitrin) | 100 | 250 | 0.9987 | 400-7500 |
| Rutin  (Quercetin 3-*O*-rutinoside) | 120 | 300 | 0.9990 | 300-6000 |
| Hyperoside  (Quercetin 3-*O*-galactoside) | 150 | 200 | 0.9988 | 500 - 15000 |
| Luteolin-7-*O*-glucoside | 50 | 80 | 0.9990 | 150-15000 |
| Isoquercetin | 150 | 275 | 0.9989 | 400 - 16000 |
| Kaempferol–3-*O*-rutinoside | 60 | 125 | 0.9990 | 125-50000 |
| Isorhamnetin-3-*O*-rutinoside (Narcissoside) | 100 | 200 | 0.9993 | 400-16000 |
| Isorhamnetin-3-*O*-glucoside | 80 | 150 | 0.9996 | 250-10000 |
| Quercitrin | 75 | 150 | 0.9989 | 250-20000 |
| Apigenin 7-*O*-glucoside | 20 | 40 | 0.9987 | 100-5000 |
| Naringenin 7-*O*-glucoside | 100 | 175 | 0.9992 | 250-25000 |

**Supplementary Figure S1.** LC-MS/MS chromatogram of phenolic compounds found in *Ceratophyllum demersum* L. ethanolic extract.

**
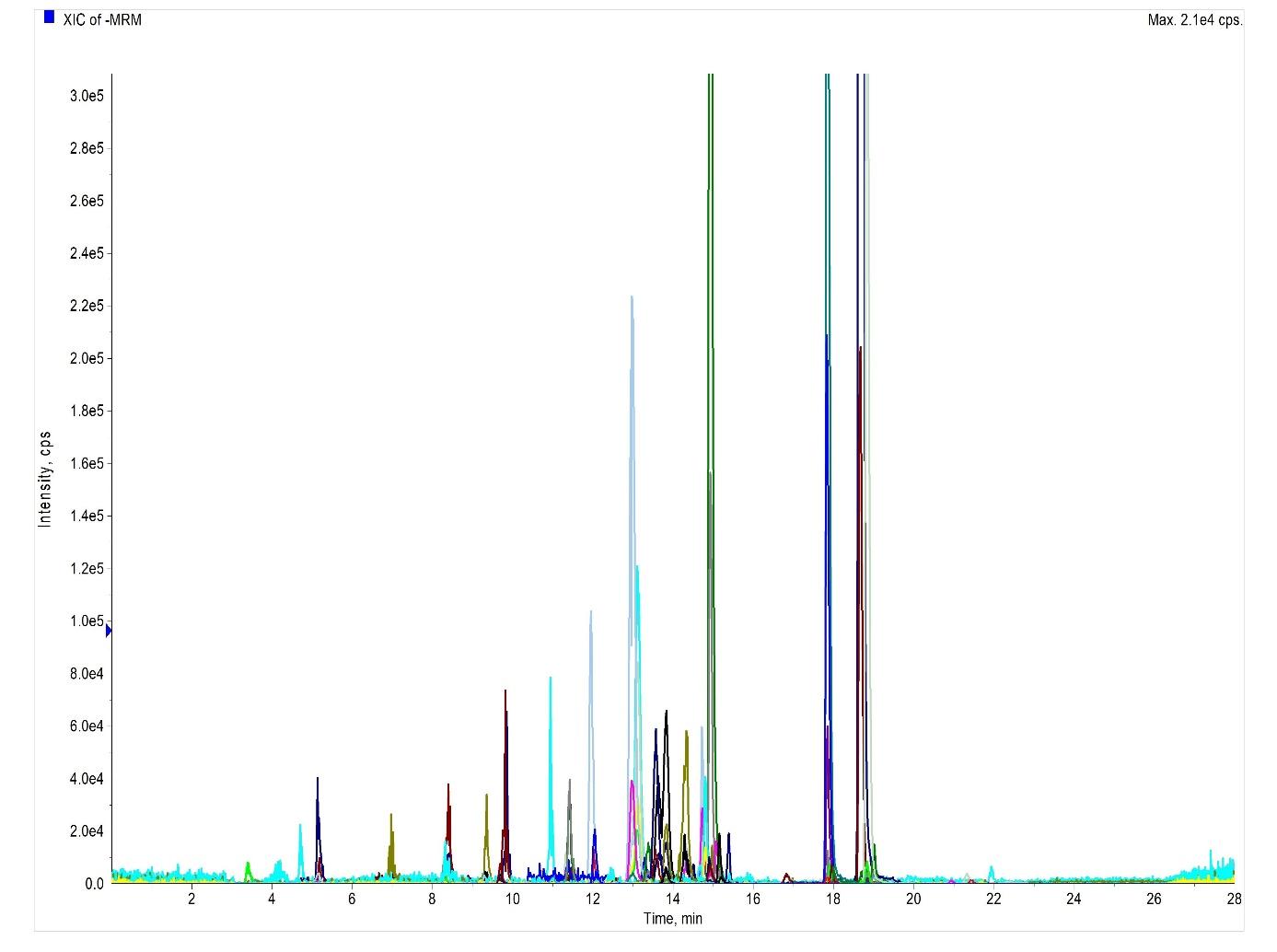
**

**Supplementary Figure S2.** LC-MS/MS-MRM chromatograms of flavonoid aglycones identified in *Ceratophyllum demersum*
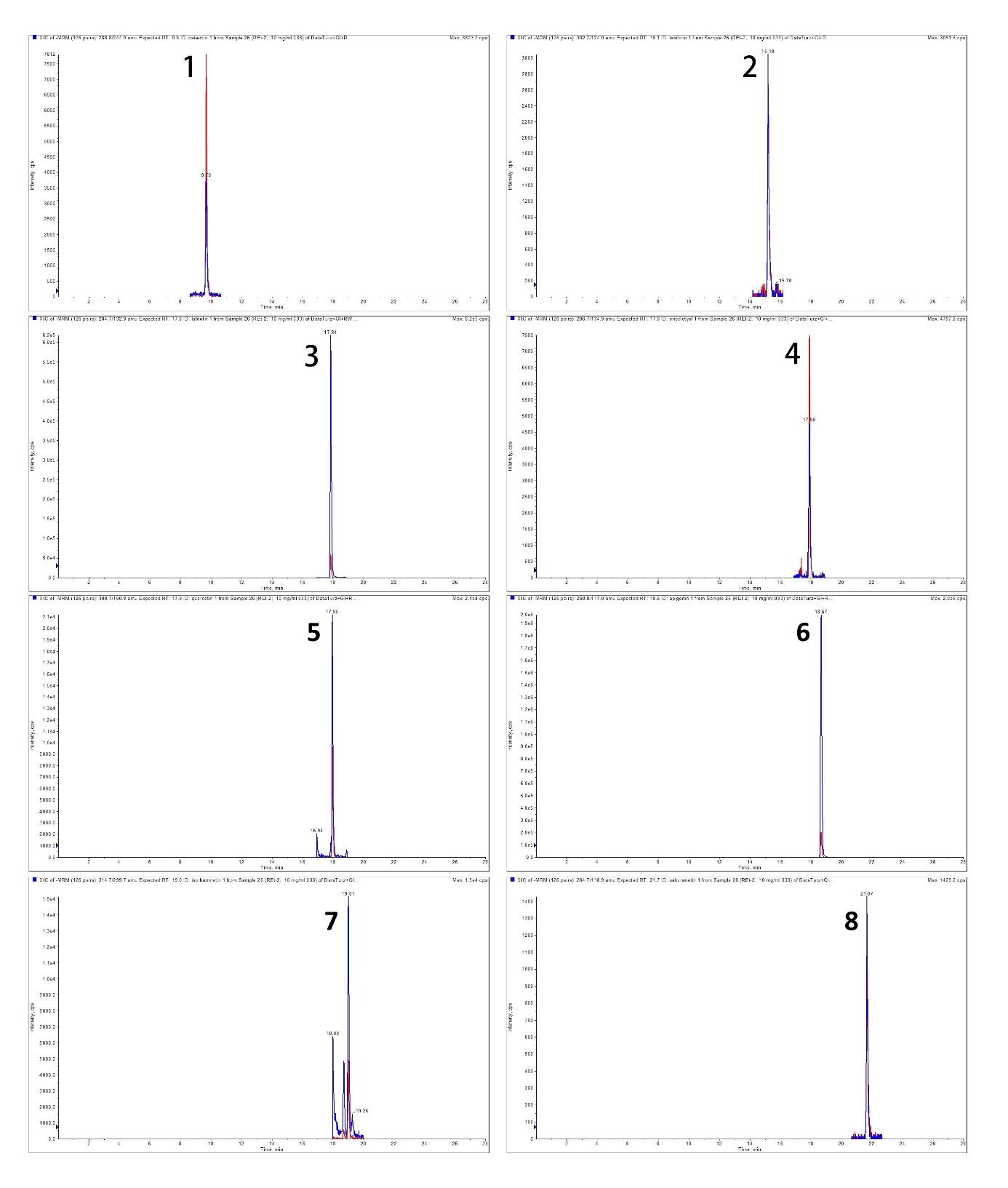
 L. ethanolic extract; 1 – catechin, 2 – taxifolin, 3 – luteolin, 4 – eriodictyol, 5 – quercetin, 6 – apigenin, 7 – isorhamnetin, 8 – sakuranetin.
